# The Potential of Fecal Volatile Organic Compound Analysis for the Early Diagnosis of Late-Onset Sepsis in Preterm Infants: A Narrative Review

**DOI:** 10.3390/s24103162

**Published:** 2024-05-16

**Authors:** Rimke R. de Kroon, Nina M. Frerichs, Eduard A. Struys, Nanne K. de Boer, Tim G. J. de Meij, Hendrik J. Niemarkt

**Affiliations:** 1 Department of Pediatric Gastroenterology, Emma Children’s Hospital, Amsterdam Gastroenterology Endocrinology Metabolism Research Institute, Amsterdam University Medical Center, University of Amsterdam, Meibergdreef 9, 1105 AZ Amsterdam, The Netherlands; 2 Amsterdam Reproduction and Development Research Institute, Amsterdam University Medical Center, University of Amsterdam, Meibergdreef 9, 1105 AZ Amsterdam, The Netherlands; 3 Department of Laboratory Medicine, Amsterdam University Medical Center, Location AMC, Meibergdreef 9, 1105 AZ Amsterdam, The Netherlands; 4 Department of Gastroenterology and Hepatology, Amsterdam Gastroenterology Endocrinology Metabolism Research Institute, Amsterdam University Medical Center, Vrije Universiteit Amsterdam, De Boelelaan 1117, 1081 HV Amsterdam, The Netherlands; 5 Department of Neonatology, Maxima Medisch Centrum, De Run 4600, 5504 DB Veldhoven, The Netherlands; 6 Department of Electrical Engineering, TU Eindhoven, Eindhoven University of Technology, Postbus 513, 5600 MB Eindhoven, The Netherlands

**Keywords:** neonatology, preterm infants, late-onset sepsis, volatile organic compounds, biomarker, detection, non-invasive diagnostics, electronic nose, intestinal microbiota

## Abstract

Early diagnosis and treatment of late-onset sepsis (LOS) is crucial for survival, but challenging. Intestinal microbiota and metabolome alterations precede the clinical onset of LOS, and the preterm gut is considered an important source of bacterial pathogens. Fecal volatile organic compounds (VOCs), formed by physiologic and pathophysiologic metabolic processes in the preterm gut, reflect a complex interplay between the human host, the environment, and microbiota. Disease-associated fecal VOCs can be detected with an array of devices with various potential for the development of a point-of-care test (POCT) for preclinical LOS detection. While characteristic VOCs for common LOS pathogens have been described, their VOC profiles often overlap with other pathogens due to similarities in metabolic pathways, hampering the construction of species-specific profiles. Clinical studies have, however, successfully discriminated LOS patients from healthy individuals using fecal VOC analysis with the highest predictive value for Gram-negative pathogens. This review discusses the current advancements in the development of a non-invasive fecal VOC-based POCT for early diagnosis of LOS, which may potentially provide opportunities for early intervention and targeted treatment and could improve clinical neonatal outcomes. Identification of confounding variables impacting VOC synthesis, selection of an optimal detection device, and development of standardized sampling protocols will allow for the development of a novel POCT in the near future.

## 1. Introduction

Prematurity (gestational age (GA) < 37 weeks) remains the leading cause of death among children under five years of age [1]. Despite improved obstetric and neonatal care, preterm infants remain susceptible to diseases related to organ immaturity such as late-onset sepsis (LOS), defined as sepsis occurring after 72 h of life [2]. LOS occurs in 20 to 30% of neonatal intensive care unit-admitted preterm infants and has a mortality rate of 13 to 19%, with survivors facing impaired growth and neurodevelopmental outcomes [3,4,5,6,7]. Early recognition of LOS is critical but remains challenging due to nonspecific clinical symptoms, which may lead to delayed initiation of antibiotic treatment [8]. Moreover, indiscriminate empirical treatment can result in antibiotic resistance and disrupted gut microbiota, which is associated with short- and long-term complications, including necrotizing enterocolitis (NEC), atopy, and asthma [9,10,11,12,13,14,15,16,17].

LOS is associated with preclinical gut microbiota and metabolome alterations and the gastrointestinal (GI) tract appears to be an important reservoir for LOS pathogens [18,19,20,21,22,23,24]. Increased abundance of several common LOS pathogens, including *Escherichia coli*, *Klebsiella pneumoniae*, *Serratia marcescens*, and *Enterococcus faecalis*, has been demonstrated in the preterm gut up to two weeks before clinical suspicion of LOS compared to healthy controls [19,25,26]. Strain-resolved analyses unveiled that the bacterial strains identified in the preclinical fecal samples were genetically similar to the blood culture isolate in 27 to 75% of the infants who developed LOS [19,26]. Specific gut microbiome signatures before LOS have also been described, varying from an increased abundance of *Bacilli* and lack of anaerobic bacteria to a decreased abundance of *Bifidobacterium*. Additionally, infants with LOS appear to have decelerated development of microbial diversity [20,21,23]. Untargeted metabolomic profiling in fecal samples revealed distinct functional profiles in infants with LOS on the day of diagnosis compared to controls [20]. Moreover, enteral therapies that influence microbiota composition and function, such as probiotics and lactoferrin, may mitigate LOS development [27,28,29,30,31], underlining the gut’s role in LOS pathogenesis. It is hypothesized that the altered development of the preterm intestinal microbiota in combination with an immature gut lining accommodates the overgrowth and translocation of potential pathogenic bacterial strains [25,27]. These findings highlight the potential for intestinal microbiota and metabolome analysis as non-invasive biomarkers for early diagnosis of LOS.

Novel strategies to diagnose gut microbiome-related diseases at an early stage have gained scientific traction over the past decade. One of these approaches is the detection of volatile organic compounds (VOCs) in human bodily secretions, which reflect a complex interplay between the host, the environment, and microbiota. VOC detection has emerged as a promising approach for early disease detection of, for example, diabetes [32,33], diseases of the GI tract and liver [34,35,36], various types of cancer [37], and infectious diseases [38]. An array of devices equipped with different chemical-detection technologies have been utilized to identify disease-associated VOCs, often aiming to develop a rapid and non-invasive point-of-care test (POCT) [39].

This review provides a state-of-the-art overview of the detection of VOCs as a tool for early diagnosis of LOS in preterm infants, focusing primarily on fecal VOCs. First, we will elaborate on various processes in the human body, with a focus on the GI tract, that influence VOC synthesis and provide an overview of the technologies capable of VOC detection. Subsequently, we will explore species-specific VOC profiles for common LOS pathogens, followed by an assessment of the current literature available on VOC detection aiding the early diagnosis of LOS. By careful deliberation of the available literature, we aim to identify the opportunities and challenges that lie within the development of a POCT for clinical practice.

## 2. Volatile Organic Compounds Reflect a Complex Interplay between the Human Host, the Environment, and the Microbiome

VOC analysis is of specific interest to a wide variety of industries as they are produced by living organisms, including plants, humans, animals, and microorganisms. VOCs are small, low-molecular-weight carbon-based molecules (<300 Da), belonging to various chemical classes (e.g., alcohols, aldehydes, ketones, hydrocarbons, acids, and esters). At room temperature, VOCs have a high vapor pressure correlating with a relatively low boiling point [40]. Due to their small size and simple chemical composition, VOCs are easily produced compared to more complex metabolites [41]. In humans, VOC profiles serve as indicators of physiologic metabolic processes but also reflect pathophysiologic changes, immune responses, host–environment, and host–microbe interactions. VOCs that are generated at the cellular level can transfer into the bloodstream and subsequently be exhaled or excreted in feces or urine [40]. A study compiling a compendium of all VOCs emitted in the healthy adult human body demonstrated that 369 VOCs were detected in blood, 1488 in exhaled breath, 433 in feces, 290 in human milk, 549 in saliva, 196 in semen, 623 in skin, and 444 in urine. Various VOCs appear in multiple matrices, while these matrices also, notably, contain unique compounds [42]. Researchers have also aimed to characterize the fecal VOC composition of healthy preterm infants [43,44,45]. Frau et al. (2021) described a sparse fecal volatile metabolome in the days after birth with a predominance of acetic acid and aldehyde in 51 preterm infants (GA < 32 weeks) [43]. A marked change occurred in VOC composition with the introduction of enteral feeding and a rise in the number of VOCs was observed between day 5 and day 10 after birth, indicating that the metabolome changes in the early life of preterm infants [43].

A plethora of VOCs is produced in the GI tract, harboring a complex and dynamic ecosystem of bacteria, fungi, and viruses [46]. Gut bacteria and fungi produce VOCs, which are capable of interfering with neighboring microbial species and the human host [41,47]. Microbial VOCs (mVOCs) are produced during primary metabolism, for example during the bacterial breakdown of amino acids. Additionally, mVOCs are produced as byproducts of secondary metabolism, for example as a result of the oxidation of glucose [48]. In microbial communication, specific mVOCs are known to play an important role, while, importantly, for the majority of mVOCs, no specific function has yet been identified [49]. One of the main processes in the GI tract that facilitates mVOC synthesis is the bacterial fermentation of non-starch polysaccharides [41]. Specific bacterial characteristics, such as antibiotic resistance, have been demonstrated to influence VOC profiles. Although a limited number of studies have been conducted on this topic, some bacteria can successfully be differentiated based on their VOC profiles which correlate with their antibiotic sensitivity [50]. mVOCs capable of inducing tolerance against microbial agents have been identified: for example, indole, an important mVOC associated with *E. coli*, which is believed to play a role in the development of multidrug tolerance through upregulations of specific antibiotic resistance-related genes [51,52]. In addition to physiologic metabolic processes and mVOC production, inflammatory processes in the gut also produce a variety of VOCs. Oxidative stress results in the production of reactive oxygen species, which contribute to the production of VOCs (e.g., ethane, pentane, methylate alkane). All of these processes are measurable as alterations in the fecal VOC profile [53].

Fecal VOC profiles are also heavily influenced by host–environmental factors, including diet, lifestyle, and medication [54,55,56,57]. A limited number of studies on this topic have been conducted in infants [55,56,57]. In 31 healthy term infants, a correlation of fecal VOC profiles to enteral feeding type (human milk versus formula feeding) and center of birth was demonstrated [56]. In addition, the two most common microbiome enterotypes within this cohort could be successfully discriminated with 88% accuracy using only two electronic nose (eNose) sensors [56]. In a cohort with 58 preterm infants (GA < 30 weeks), the influence of GA and mode of delivery on fecal VOC profiles, as detected with eNose, was assessed [55]. Although no significant differences could be demonstrated between the two groups based on the mode of delivery and gestational age (24–26 weeks versus 27–29 weeks), stratification according to postmenstrual age at the time of sample collection rather than GA was hypothesized to influence detected VOC patterns, similar to findings from microbiota studies [55]. In contrast, the enteral feeding type does significantly influence fecal VOC profiles [57,58]. Comparison of fecal VOC profiles in healthy preterm infants (GA < 30 weeks) receiving primarily human milk as opposed to formula revealed a statistically significant difference with modest discriminatory accuracy (AUC [95%CI]: 0.64 [0.51–0.77], *p* = 0.04). These changes are, at least in part, mediated by related changes in gut microbial diversity and the available substrates associated with the different feeding types. VOCs derived from formula milk contain, predominantly, alcohols, aldehydes, and ketones, in contrast to VOCs derived from human milk, which contain, predominantly, secondary oxidation products and terpenes [57]. While it has been established that environmental factors can affect fecal VOC profiles, the precise metabolites causing this change are still largely unknown. Concurrent analysis with GC-MS could identify and quantify these individual metabolites, which may provide additional insight into the underlying mechanisms.

## 3. Analytical Devices for the Detection and Analysis of Volatile Organic Compounds

**Figure 1:**
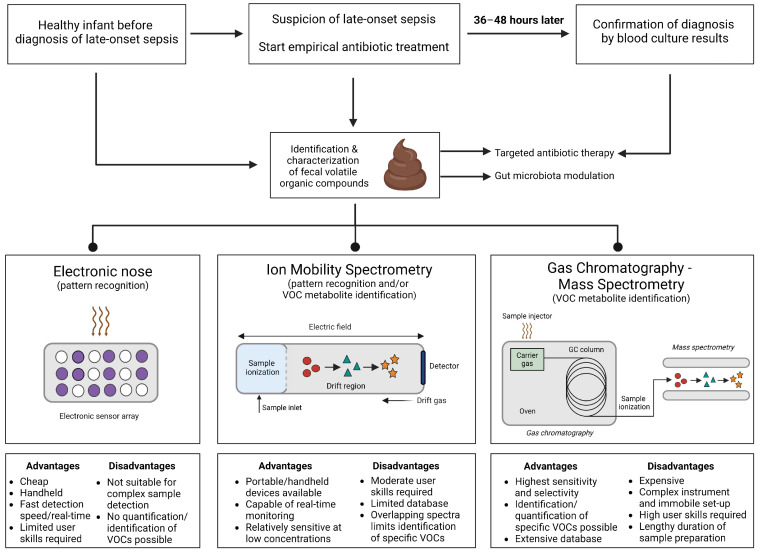
**Simplified overview of analytical techniques used for volatile organic compound analyses of preterm infants with late-onset sepsis with their advantages and disadvantages.** Diagnosis of late-onset sepsis (LOS) in a preterm infant requires adequate assessment of clinical symptoms, laboratory findings, including C-reactive protein and hematological parameters, and blood culture results. Recognition of LOS remains challenging, resulting in unnecessary antibiotic treatment or delayed initiation of targeted therapy. Identification and characterization of fecal volatile organic compounds (VOCs) may aid in early diagnosis of LOS in preterm infants and guide the initiation of targeted antibiotic treatment or gut microbiota modulation (e.g., fecal microbiota transplant, probiotics). Fecal VOC profiles can be assessed using an electronic nose, ion mobility spectrometry, and/or gas chromatography–mass spectrometry. These approaches require different detection techniques, and have different advantages, and disadvantages, as summarized in Figure 1. Figure created with Biorender.com. Accessed on 20 March 2024. Abbreviations: VOCs, volatile organic compounds; GC, gas chromatography.

Various analytical methods are available for the detection and/or quantification of VOCs in human samples, including blood, feces, and exhaled breath. To utilize VOCs for the detection of human states (e.g., healthy versus diseased) in clinical practice, these methods must be able to detect VOCs in the headspace of highly diverse substrates. These analytical devices can be divided into two main categories, chemical analytical techniques and pattern-based techniques, with diverse characteristics and varying potential for the development of a POCT for early detection of LOS (Figure 1).

The golden standard for separation, detection, and identification of VOCs remains gas chromatography–mass spectrometry (GC-MS) [59,60,61,62]. GC technologies include two-dimensional GC-MS (GCxGC-MS) or may be integrated with time-of-flight (TOF)-MS, allowing for the separation of complex mixtures [63]. Proton transfer reaction–mass spectrometry (PTR-MS) or selected ion flow-tube mass spectrometry (SIFT-MS) serve as alternatives for GC-MS [64,65,66]. While GC-MS allows for the comprehensive identification of VOCs, drawbacks include extensive sample pre-treatment, high user skill level, significant costs, and set-up immobility. However, GC-MS will, unarguably, play a prominent role in translating findings from more advanced approaches to clinical practice, through the identification of distinctive disease-associated VOCs to train more practical pattern-based devices.

An alternative to GC-MS is ion-mobility spectrometry (IMS). IMS devices are often portable and can yield fast and real-time results [67]. A well-studied example device for detecting and quantifying VOCs is field asymmetric ion mobility spectrometry (FAIMS), which has contracted attention due to its increased sensitivity and real-time discrimination of VOC profiles at a lower cost than other IMS devices [68]. While IMS devices require a relatively high user skill level, they can be used on their own as screening tools to assess volatile patterns in a bedside setting or they can be coupled with GC to provide multi-dimensional separation.

eNose devices, which rely on pattern recognition, perhaps have the highest clinical potential to identify infants at risk for LOS. eNoses are small, inexpensive, and easy to operate with limited training, and are therefore ideal in a bedside setting [63,69,70]. The typical eNose is composed of an array of 8–32 chemical sensors, adapted to sense different chemical groups, and can be trained to recognize specific diseases [71]. Besides instruments that utilize conducting polymers like the Cyranose 320 (Cyranose Sciences Sensor Technology), a variety of other techniques are available, including surface acoustic wave, quartz crystal microbalance, and metal oxide semiconductors. When selecting an appropriate approach, the sensor sensitivity, detection mechanism, and response time of the eNose device should be considered [72]

Various studies have focused on the characterization of VOC profiles in neonatal stool samples of both healthy infants and infants with comorbidities (e.g., LOS, NEC, bronchopulmonary dysplasia) (Supplementary Table S1). The majority of studies have utilized eNose devices, specifically Cyranose 320, for fecal volatilome analysis [55,56,57,73,74,75,76,77]. To a lesser extent, studies in neonatal cohorts have utilized GC-MS-based [43,45,58,78,79] and IMS-based approaches [44,79,80,81].

## 4. Species-Specific Volatile Organic Compound Profiles for Common Pathogens of Late-Onset Sepsis

A VOC-based POCT must be able to detect LOS-associated profiles or even profiles of specific bacterial strains that cause LOS as a result of gut translocation. Common causative pathogens are Gram-positive bacteria (~45–85%), including coagulase-negative *staphylococci* (CoNS) and *Staphylococcus aureus*, often associated with catheter-related bloodstream infections, and Gram-negative bacteria (~15–25%), including *E. coli*, *K. pneumoniae*, and *Pseudomonas aeruginosa* [82]. A systematic review by Bos et al., including studies utilizing a variety of techniques in both in vitro and ex vivo models, focused on elucidating VOC profiles for etiological agents of adult sepsis, which overlap to some extent with LOS pathogens [83]. Although many VOCs were present in relation to multiple strains, discriminatory VOCs were identified for three pathogens: isovaleric acid and 2-methyl-butanal (*S. aureus*), 1-undecene, 2,4-dimethyl-1-heptane, 2-butanone, 4-methyl-quinazoline, hydrogen cyanide, and methyl thiocyanide (*P. aeruginosa*), and methanol, pentanol, ethyl acetate, and indole (*E.coli*) [83].

In a recent review, VOC analyses of common bacterial pathogens causing bloodstream infections and other infectious diseases were discussed [84]. A list was compiled of 93 VOC metabolites associated with common infectious agents discovered in in vitro experiments. VOCs were reported if they were put forward as a discriminatory metabolite in two or more unrelated research experiments focusing on the same bacterial pathogen. Similar to the previous study, the majority of those 93 observed VOCs were present in relation to more than one bacterial strain, explained by bacterial metabolic pathway similarities. For example, 78 different VOCs were associated with *P. aeruginosa*, 64 with *E. coli*, 42 with *K. pneumoniae*, and 40 with *S. aureus* [84]. Focusing on species-specific VOCs, 2-tridecanone and 2-pentadecanone, 1-octanol, and 1-hexadecanol were exclusively produced by *E. coli.* While indole, a product of tryptophan metabolism, is often identified as a characteristic VOC for *E. coli*, it is not unique to *E. coli* since it is also released by *S. aureus*, *K. pneumoniae*, and *P. aeruginosa* [84]. For *S. aureus*, isovaleric acid, ethyl 2-methylbutyrate, and acetoin were identified as possible biomarkers. The latter two were also produced by other bacterial strains, while isovaleric acid was the only distinctive VOC metabolite for *Staphylococcal* spp. As many as 19 characteristic VOCs have been described for *P. aeruginosa*, including oxygenated compounds, cyanide compounds (e.g., hydrogen cyanide, methyl thiocyanate), nitrogen-containing compounds, and a large range of hydrocarbons (e.g., decane, 1-nonene). For *K. pneumoniae*, a variety of produced VOCs could be identified, specifically ketones; however, none of these were unique to this pathogen [84].

**Figure 2:**
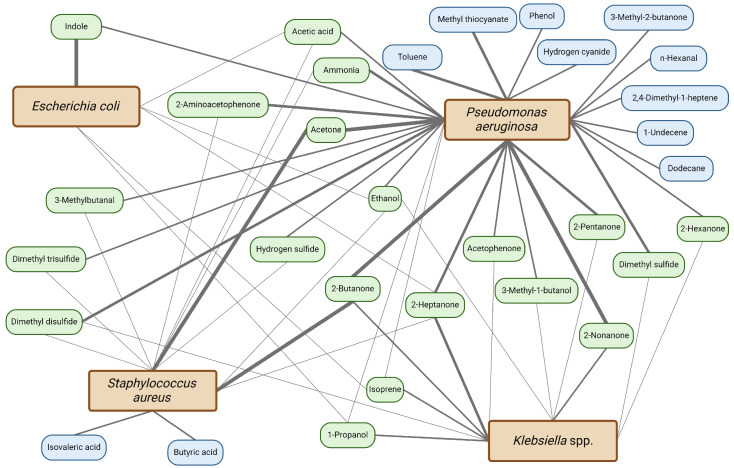
**Overview of volatile organic compounds produced by clinical isolates of common late-onset sepsis pathogens *Escherichia coli*, *Staphylococcus aureus*, *Klebsiella* spp. and *Pseudomonas aeruginosa*.** Depicted are four common LOS pathogens (orange) connected to the volatiles they produce. Only the VOCs that have been described in two unrelated research experiments focusing on the same pathogen have been included in this overview. The metabolites in blue represent the VOCs that are uniquely produced by one pathogen, and the metabolites in green are produced by two or more of the pathogens included in this overview. The width of the lines indicates the number of studies that have designated that specific metabolite as discriminatory for the pathogen: a thicker line indicates more studies. To note, only the in vitro studies that have utilized clinical isolates, ex vivo studies, and in vivo studies are displayed. References can be found in Supplementary Table S2. Figure created with Biorender.com. Accessed on 20 March 2024.

Figure 2 provides an overview of the VOCs that are produced by clinical isolates of the common LOS pathogens *E. coli*, *S. aureus*, *K. pneumoniae*, and *P. aeruginosa* from in vitro, ex vivo, and in vivo experiments [80,85,86,87,88,89,90,91,92,93,94,95,96,97,98,99,100]. Only the VOCs that have been described in two unrelated research experiments for the same pathogen are displayed (Figure 2). A list of all VOCs produced by the clinical isolates can be found in Supplementary Table S2. In the studies that measure VOC production from clinical isolates, *P. aeruginosa* and *S. aureus* produce unique metabolites; however, *E. coli* and *Klebsiella* spp. do not appear to produce distinct metabolites. In addition, Figure 2 demonstrates that many of the discovered metabolites overlap between species.

While bacterial VOC profiles have been studied most extensively, fungal pathogens also produce VOCs. *Candida* spp. can cause invasive infections in preterm infants [101]. Known volatiles of *Candida* spp. include ethanol, acetaldehyde, acetone, methanethiol, 2-butenal, isoamyl alcohol, phenethyl alcohol, and cyclohexane [102]. Specifically, for the characterization of fecal VOC profiles, these findings are of interest since a part of systemic candidiasis cases might also be gut-derived similar to bacterial sepsis [101].

Moreover, various confounding factors can influence mVOC patterns in in vitro models, including storage conditions, bacterial growth, culture media, and the presence of multiple pathogens in a culture, while host and environment interactions cannot be measured [84]. The extensive number of confounding factors influencing mVOC patterns could explain the differences between the key VOCs displayed in Figure 2, based solely on clinical isolates of common LOS pathogens, as opposed to VOCs identified in the previously mentioned reviews [83,84]. Due to the overlapping metabolites and/or profiles, identification of specific bacterial or even fungal strains in a more complex sample, such as feces, will be challenging.

## 5. Distinct Volatile Organic Compound Profiles Associated with Late-Onset Sepsis

**Figure 3:**
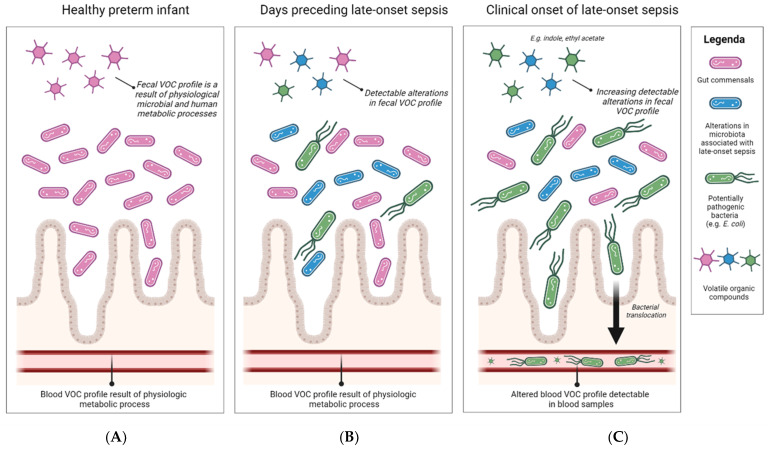
**Proposed mechanism of volatile organic compound detection in stool samples of preterm infants with late-onset sepsis.** (**A**) In healthy preterm infants, the fecal volatile organic compound (VOC) profile results from physiologic microbial and host metabolic processes. (**B**) In the days prior to the clinical onset of late-onset sepsis, alterations in microbial composition result in detectable alterations in fecal VOC profile. (**C**) Upon diagnosis of late-onset sepsis, increasing amounts of pathogenic bacteria have translocated across the compromised gut barrier, resulting in a systemic response. Alterations in VOC profiles due to human inflammatory processes as well as microbial VOCs can be detected in stool samples as well as in blood samples. Figure created with Biorender.com. Accessed on 20 March 2024. Abbreviations: *E. coli*, *Escherichia coli*; VOCs, volatile organic compounds.

Almost all VOC studies concerning LOS in preterm infants utilized stool samples with only one study using another substrate (Table 1, Figure 3). A cohort of 28 intubated preterm infants (GA < 37 weeks) of which 8 infants were diagnosed with a blood culture-proven bloodstream infection used an eNose device on tracheal aspirates to successfully identify the infants diagnosed with a bloodstream infection, regardless of tracheal aspirate culture (Table 1) [103]. However, tracheal aspirates can only be obtained from intubated infants and are not suitable as a non-invasive biomarker for infants not requiring intubation.

In a proof of principle study, a Cyranose 320 was used to assess the fecal VOC profiles in preclinical samples of LOS patients and matched healthy controls (GA < 30 weeks) (Table 1). The majority of LOS episodes were caused by CoNS, followed by *S. aureus*, and *E. coli*. Infants with LOS could be successfully identified at one, two, and three days before LOS based on their fecal VOC profile [74]. In a larger follow-up cohort study, LOS patients were compared with healthy matched controls using FAIMS (Table 1) [81]. The *E. coli* LOS cases could be identified with the highest accuracy at all three time points prior to clinical suspicion, followed by *S. aureus* and *S. epidermidis* [81]. FAIMS was also utilized in a cohort of non-catheter-related LOS cases [76]. The preclinical fecal VOC profiles aided in the distinction between cases and controls with varying predictive values. Of note, when analyzing the *S. epidermidis* cases one day prior to clinical suspicion, an area under the curve (AUC) of 0.95 could be obtained [76]. These findings exceed the AUC of 0.63 found in the previous study by Berkhout et al. [81], which, in addition to non-catheter-related *S. epidermidis* cases, also included cases with presumed catheter-related etiology (Table 1).

To identify unique metabolites, preclinical fecal samples were analyzed using two approaches, GC-IMS and GC-TOF-MS [79]. Gram-negative LOS cases could be discriminated at 2 and 3 days before onset when assessed with GC-IMS and at 1 day before onset when assessed with GC-TOF-MS (Table 1). Using GC-IMS, LOS episodes caused by *E. coli* could significantly be distinguished from controls at one and two days before onset. To a lesser extent, Gram-positive LOS episodes could be discriminated from healthy controls one day before onset using GC-IMS, followed by CoNS episodes. GC-TOF-MS analysis identified 15 unique distinctive metabolites. For Gram-negative pathogens, acetate, ethyl 2-(methylamino)acetate, ethyl 2-hydroxy propanoate, prop-1-ene, butane-2,3-dione, and 2,2,4,4-tetramethylpentane were indicative, while for CoNS cases, heptanal was most indicative [79]. One of the Gram-negative discriminatory VOCs was ethyl acetate, which also came forward in previous MS studies on *E. coli* sepsis, similar to the well-studied indole [83]. However, the current study did not find indole as a discriminatory metabolite [79]. A comparison of fecal VOC profiles between adults and infants demonstrated that indole could be identified in 100% of adult fecal samples (50 samples collected over two weeks from 10 adults) in contrast to 33% of neonatal samples (36 samples collected over two weeks from seven neonates). Of the included infants, four infants lacked indole completely [45]. Many knowledge gaps remain regarding the mechanism behind the production of indole and how gut bacteria regulate indole signaling. Most likely, these mechanisms and environmental factors, such as dietary intake, which impact indole production differ between adults and infants and should be taken into account when assessing LOS-associated or even species-specific fecal VOC profiles.

## 6. Opportunities and Challenges of Clinical Application of Volatile Organic Compound Analysis for Early Diagnosis of Late-Onset Sepsis

**Figure 4:**
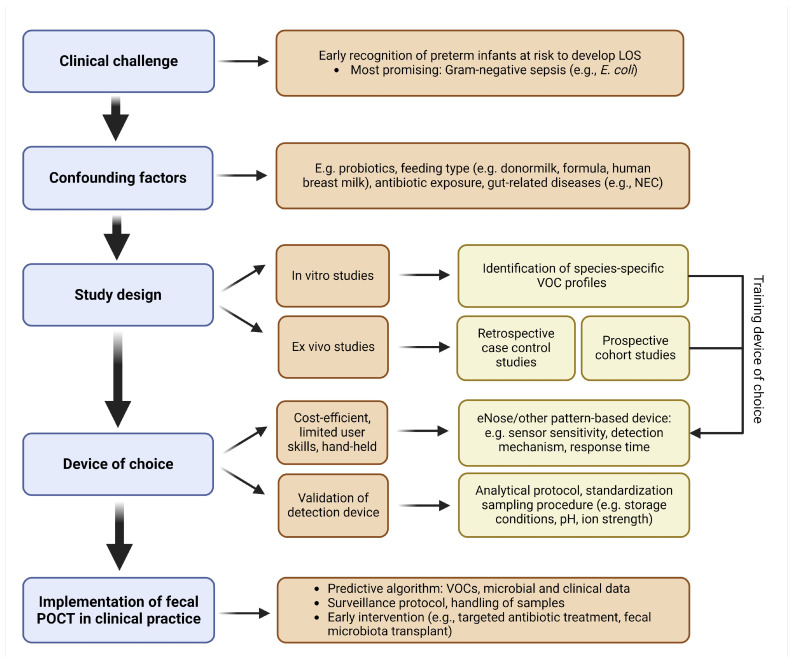
**Flowchart guiding the development of a fecal point-of-care test for the early detection of late-onset sepsis in preterm infants**. Figure created with Biorender.com. Accessed on 20 March 2024. Abbreviations: LOS, late-onset sepsis; *E. coli*, *Escherichia coli*; NEC, necrotizing enterocolitis; VOC, volatile organic compounds; eNose, electronic nose; POCT, point-of-care test.

An increasing body of evidence points towards a pivotal role for the gut microbiome in the pathogenesis of LOS in preterm infants [22], suggesting that identification of alterations in the gut microbial or metabolic composition and their function could serve as a promising tool for early diagnosis or even prediction of infants at risk to develop LOS. In this next section, the opportunities and challenges that lie within the clinical application of VOC detection are explored (Figure 4).

Characterization of VOC patterns in stool samples up to three days preceding the onset of clinical symptoms distinguished patients with LOS from healthy controls with varying predictive values [74,76,79,81]. A higher predictive value was associated with a shorter time interval to clinical onset [74], possibly due to alterations in the host state metabolism, as well as the increasing abundance or metabolic activity of LOS causative agents in the gut prior to clinical disease onset (Figure 3) [25]. Fecal VOC profiling was most promising for the identification of Gram-negative cases, followed by *S. aureus* cases [79,81], which could be attributed to the association of *S. aureus* with catheter-related or central line-related infections rather than a gut-derived origin [104,105]. CoNS episodes were least accurately identified, most likely due to having a similar etiology as *S. aureus* LOS episodes. These findings are underlined by the increased AUC when all catheter-associated CoNS episodes are excluded from the analysis [76].

The main studied substrate was fecal samples. The most important benefit of stool samples is the non-invasive nature as frequent blood sampling is correlated to anemia [106] as well as altered brain structure and increased stress hormone levels as a result of repeated procedural pain [107,108]. Another benefit of studying VOC patterns in fecal samples is the potential of identifying high-risk patients prior to developing symptoms, as alterations in blood VOC profile are most likely to be identified after bacterial translocation to the bloodstream has already occurred. The use of fecal VOC profiling seems most promising for episodes caused by Gram-negative pathogens that typically have their origin in the gut, such as the common Gram-negative LOS pathogen *E. coli* [27]. In clinical practice, LOS caused by *E. coli* is feared for short-term complications, such as meningitis, long-term morbidities, and high mortality rates. Based on the currently available literature, future research should focus on developing a fecal VOC POCT capable of recognizing Gram-negative LOS or even *E. coli*-specific patterns.

To develop a sensitive, specific, and robust POCT, multiple challenges will be encountered. While in vitro, ex vivo, and in vivo studies have aimed to demonstrate species-specific VOC profiles, translation of these findings to clinical practice is hampered due to overlapping profiles between pathogens. Moreover, in vitro studies do not reflect the complex interplay between the gut microbiome, the host metabolic processes, and environmental factors [88,100,109]. So far, only one retrospective case-control ex vivo study attempted to identify VOCs associated with Gram-negative LOS [79]. Furthermore, when identifying VOCs associated with bacterial species or specific bacterial groups, such as Gram-negative pathogens, the impact of interstrain diversity on VOC profiles should be taken into account. For example, Fitzgerald et al. (2020) identified 1-decanol and 2-tridecanol as distinctive volatiles for one of the studied *E. coli* strains (DSM30083), while 1-hexadecanol was only present in a second studied *E. coli* strain (DSM105372) [110]. Further research should focus on elucidating a core set of VOCs that are not influenced by strains but that reflect the common LOS pathogens on a species level. These findings together with in vitro results can be used as a starting point to design studies utilizing targeted metabolomics and subsequently training eNose devices to recognize infants at risk for Gram-negative LOS.

When developing a fecal VOC-based POCT, the interindividual variability and impact of environmental factors, medical interventions, or other host states should be taken into account. The confounding factors on the fecal volatilome in healthy preterm infants warrant further investigation, including enteral supplementation with probiotic strains, which is hypothesized to protect against NEC and potentially also LOS [111]. As of now, no studies have taken the possible effect of probiotic supplementation on the fecal VOC composition into account. Other interventions such as donor milk administration or antibiotic exposure could also impact VOC profiles. Of note, GI diseases such as NEC are also associated with alterations in fecal VOC profiles and could interfere with the profiles associated with LOS [58,73]. Determination of disease-specific VOC profiles is necessary before the deployment of a VOC-based POCT in preterm care.

The device of choice for VOC detection, for example, an eNose device, must be capable of accurately distinguishing patients with LOS from healthy controls while being cost-efficient and requiring limited user skill. Due to the evaporative nature of VOCs, analysis should ideally take place right from the diaper [44], or immediately after sampling at the bedside to ensure reliable results. Potentially, a bedside VOC analyzer could even be built in the incubator [112]. A consensus should be reached on applied techniques and standardization of sampling procedures in large-scale validation studies. Various factors, including sample mass, sample water content, sample temperature, duration of storage at room temperature, and number of freeze–thaw cycles, have been demonstrated to affect the observed fecal volatile profile as measured by eNose [75]. Storage conditions can affect the availability of VOCs, in addition to the pH value and ionic strength of the samples. The type of fecal sample (e.g., fecal swab versus stool sample) also influences the detected VOC profiles [75]. Taking into account the effect of local research protocols on fecal VOC profiles, future research should utilize comparable protocols limiting the risk of bias caused by environmental factors. Of note, optimal sampling and storage conditions may differ for other techniques, such as GC-MS- and IMS-based approaches. Lastly, the use of alcohol-based hand sanitizers, which contain a variety of VOCs, should be taken into account when constructing an optimal standardized sampling protocol [113].

The implementation of a VOC-based POCT for early detection of LOS potentially has major clinical implications in the neonatal intensive care unit. Combining fecal VOC profiling with microbial analysis and clinical characteristics in a predictive machine learning model could provide the additional relevant information required for the early identification of high-risk infants. Implementation of a POCT will possibly allow early intervention, such as administration of targeted antibiotics or gut microbiota modulation (e.g., fecal microbiota transplantation, probiotics), and also decrease unnecessary empiric antibiotic initiation, which may improve neonatal health outcomes. Prospective cohort studies should be conducted to assess adequate surveillance time intervals (e.g., daily or multiple times per week) and evaluate the possibility of antibiotic stewardship and/or gut microbiota modulation in high-risk infants.

## 7. Conclusions

Future research efforts should focus on translating findings from well-monitored research settings into clinical practice. Increasing knowledge on the origin of VOCs, distinctive LOS- and species-specific VOC profiles, confounding factors, and consensus on which VOC analyzing device is most suitable for clinical use is required before successful implementation in clinical practice. In addition, standardized technical protocols are necessary to guide further research on this topic in larger (prospective) cohort studies. The development and implementation of a non-invasive point-of-care biomarker for LOS could allow for timely, targeted initiation of antibiotic treatment or gut microbiota modulation and consequently transform the landscape of LOS and care in preterm infants.

## Figures and Tables

**Table 1:** Overview of the clinical studies discussed in this review focusing on the clinical application of VOC profiling in late-onset sepsis in preterm infants.

Reference	Sample Size	GA (w)	Diagnosis	Sample Type	Timing Sampling	Analytical Method	Pathogen(s)	Test Accuracy and Proposed VOC Biomarker ^1^			
								All Pathogens Combined	CoNS Pathogens	Gram-Negative Pathogens	Other Gram-Positive Pathogens
Berkhout (2017) [71]	36 cases vs. 40 controls	<30	LOS	Stool	Up to 5 days before onset of episode	eNose (Cyranose 320)	CoNS (n = 28), *S. aureus* (n = 5), *E. coli* (n = 2), multiple pathogens (n = 1)	t-1 = 0.70 * t-2 = 0.78 * t-3 = 0.70 * t-4 = 0.61 t-5 = 0.63 t-1 to -3 = 0.67 *	N.a.	N.a.	N.a.
Berkhout (2019) [81]	127 cases vs. 127 controls	<30	LOS	Stool	Up to 3 days before onset of episode	FAIMS	CoNS (n = 67), other Gram-positive strains (n = 24), Gram-negative strains (n = 21), fungi (n = 1), multiple pathogens (n = 14)	t-1 = 0.58 t-2 = 0.61 t-3 = 0.59 t-1 to -3 = 0.56 *	Tlast = 0.56	Tlast = 0.77 * t-1 to -3 (*E. coli)* = 0.87 *	Tlast = 0.74 * t-1 to -3 (*S. aureus*) = 0.69 *
Berkhout (2020) [76]	24 cases vs. 24 controls	<30 weeks	LOS	Stool	Up to 3 days before onset of episode	FAIMS	CoNS (n = 15), *E. coli* (n = 3), *S. aureus* (n = 2), *E. faecalis* (n = 1), *S. agalactiae* (n = 1), *K. pneumoniae* (n = 1), *E. aerogenes* (n = 1)	t-1 = 0.78 * t-2 = 0.65 t-3 = 0.78 * t-1 to -3 = 0.69 *	t-1 to -3 = 0.79 * t-1 to -3 (*S. epidermidis*) = 0.95 *	N.a.	N.a.
Frerichs (2023) [79]	121 cases vs. 121 controls	<30 weeks	LOS	Stool	Up to 3 days before onset of episode	GC-IMS (n = 242)	CoNS (n = 57), other Gram-positive strains (n = 23), Gram-negative strains (n = 31), multiple pathogens (n = 8), fungi (n = 2)	t-1 = 0.63 * t-2 = 0.72 * t-3 = 0.57 t-1 to -3 = 0.70 *	t-1 = 0.60 t-2 = 0.46 t-3 = 0.55 t-1 to -3 = 0.72 *	t-1 = 0.64 t-2 = 0.81 * t-3 = 0.85 * t-1 to -3 = 0.73 *	t-1 = 0.78 * t-2 = 0.43 t-3 = 0.43 t-1 to -3 = 0.70
						GC-TOF-MS (n = 68)	CoNS (n = 9), other Gram-positive strains (n = 9), Gram-negative strains (n = 15), multiple pathogens (n = 1), fungi (n = 0)	t-1 = 0.53 t-2 = 0.60 t-3 = 0.48 t-1 to -3 = 0.77*	t-1 = 0.52 t-2 = 0.32 t-3 = 0.47 t-1 to -3 = 0.69*	t-1 = 0.82* t-2 = 0.61 t-3 = 0.73 t-1 to -3 = 0.78*	t-1 = 0.54 t-2 = 0.64 t-3 = 0.44 t-1 to -3 = 0.52
								2-methylprop-1-ene, 2-(aziridin-1-yl) ethanamine, propan-2-one, cyclopentane, methoxymethane, propan-2-ol, dichloromethane	Heptanal	Ethyl acetate, ethyl 2-(methylamino) acetate, ethyl 2-hydroxypropanoate, prop-1-ene, butane-2,3-dione, 2,2,4,4-tetramethylpentane	N.a.
Rogosch (2014) [103]	8 cases	<37 weeks	BSI	Tracheal aspirates	BSI	eNose (Cyranose 320)	Not specified	BSI vs no BSI (TA culture+): CVV 62.5% * BSI vs. no BSI (TA culture−): CVV 70% *	N.a.	N.a.	N.a.

Abbreviations: BSI, bloodstream infection; CoNS, coagulase-negative staphylococci; CVV, cross-validation value; eNose, electronic nose; FAIMS, field asymmetric ion mobility spectrometry; GA, gestational age; GC-IMS, gas chromatography–ion mobility spectrometry; GC-TOF-MS, gas chromatography coupled to time-of-flight mass spectrometry; LOS, late-onset sepsis; TA, tracheal aspirate; Tlast, last sample obtained before clinical onset of sepsis; t-1/-2/-3/-4/-5 = 1/2/3/4/5 days before clinical onset of sepsis. ^1^ Area under the curve is provided unless otherwise specified. * Significance *p* < 0.05.

## Data Availability

Data available within the article or its Supplementary Materials.

## Supplementary Materials

The following supporting information can be downloaded at: https://www.mdpi.com/article/10.3390/s24103162/s1, Table S1: Overview of applied techniques in clinical studies on detection of fecal volatilome in infants; Table S2: Overview of all the volatile organic compounds produced by clinical isolates of common late-onset sepsis pathogens *Escherichia coli*, *Staphylococcus aureus, Klebsiella* spp. and *Pseudomonas aeruginosa*. VOCs are displayed if they were found to be a discriminating volatile metabolite for that pathogen or if they were released by the pathogen.

## Abbreviations

List of acronyms and abbreviations (alphabetical)

AUC	Area under the curve
BW	Birth weight
CoNS	Coagulase-negative *Staphylococci*
eNose	Electronic nose
FAIMS	Field asymmetric ion mobility spectrometry
GA	Gestational age
GC	Gas chromatography
GC-TOF-MS	Gas chromatography coupled to time-of-flight mass spectrometry
GC-MS	Gas chromatography–mass spectrometry
IMS	Ion mobility spectrometry
LOS	Late-onset sepsis
mVOC	Microbial VOC
MS	Mass spectrometry
POCT	Point-of-care test
PTR-MS	Proton transfer reaction–mass spectrometer
SIFT-MS	Selected ion flow-tube mass spectrometer
SIRS	Systemic inflammatory response syndrome
TOF-IMS	Time-of-flight-ion mobility spectrometry
VOC	Volatile organic compound
