# Supplementary material for: The Potential of Fecal Volatile Organic Compound Analysis for the Early Diagnosis of Late-Onset Sepsis in Preterm Infants: A Narrative Review
Source: Sensors (Basel). 2024 May 16;24(10):3162. doi: 10.3390/s24103162 (PMC11124895; doi:10.3390/s24103162)
Supplement: Supplementary file 1 [file sensors-24-03162-s001.zip › sensors-2950055-supplementary.pdf]

Supplementary Table S1: Overview of applied techniques in clinical studies on detection of fecal volatilome in infants.

	Reference	Author	Year
Electronic Nose			
Cyranose 320	[55]<br>[56]<br>[57]<br>[73]<br>[74]<br>[77]	Deianova<br>Hosfield<br>El Manouni El Hassani<br>De Meij<br>Berkhout<br>Berkhout	2020<br>2020<br>2018<br>2015<br>2017<br>2018
Gas chromatography—mass spectrometry			
GC-MS	[43]<br>[45]<br>[78]	Frau<br>De Lacy Costello<br>Garner	2021<br>2008<br>2009
GC-TOF-MS	[79]	Frerichs	2023
SPME GC-MS	[58]	Probert	2009
Ion mobility spectrometry			
GC-IMS	[79]	Frerichs	2023
MCC-IMS	[44]<br>[80]	Bous<br>Bous	2023<br>2023
FAIMS	[81]<br>[76]	Berkhout<br>Berkhout	2019<br>2020

Abbreviations: eNose, electronic Nose; FAIMS, field asymmetric ion mobility spectrometry; GC-IMS, gas chromatography—ion mobility spectrometry; GC-TOF-MS, gas chromatography coupled to time-of-flight mass spectrometry; MCC-IMS, multi-capillary column—ion spectrometry; SPME GC-MS, solid phase micro-extraction gas chromatography/mass spectrometry.

Supplementary Table S2. Overview of all the volatile organic compounds produced by clinical isolates of common late-onset sepsis pathogens *Escherichia coli, Staphylococcus aureus, Klebsiella spp.*, and *Pseudomonas aeruginosa*. VOCs are displayed if they were found to be a discriminating volatile metabolite for that pathogen or if they were released by the pathogen.

Volatile organic compounds	CAS-number	*Escherichia coli*	*Staphylococcus aureus*	*Klebsiella* spp.	*Pseudomonas aeruginosa*
2-Ethylhexanol	104-76-7				[93]
(E)-1,3-Pentadiene	504-60-9			[91]	
(E)-3-Phenylprop-2-enal	14371-10-9			[97]	
1-(4-Ethylphenyl)-ethanone	937-30-4				[89]
1,1,2,2-Tetrachloroethane	79-34-5		[87]		
1,3,3-Trimethyl-(bicyclo)-heptan-2-ol	Not provided				[89]
1,4-Pentadiene	591-93-5		[95]		
1-Butanol	71-36-3			[91]	
1-Butene	106-98-9			[91]	
1-Decanol	112-30-1	[93]			
1-Octanol	111-87-5	[93]			
1-Pentanol	71-41-0		[90]		[99]
1-Phenylpropan-2-one	103-79-7			[97]	
1-Propanol	71-23-8	[87]		[87,91]	[99]
1-Undecene	821-95-4				[87,92]
1-vinyl aziridine	5628-99-9				[88]
1-α-Pinene	80-56-8				[92]
2,2,6-Trimethyloctane	62016-28-8				[92]
2,3-Butanedione	431-03-8	[96]		[91]	
2,3-Hexanedione	3848-24-6				[86]
2,3-Pentanedione	600-14-6				[86]
2,4,6-Trimethylpyridine	108-75-8				[86]
2,4-Dimethyl-1-heptene	19549-87-2				[86,98]
2,4-Dimethylfuran	3710-43-8				[86]
2,5-Dimethylpyrazine	123-32-0				[89]
2-Aminoacetophenone	551-93-9		[90]		[86,90,99]
2-Aminopyridine	504-29-0				[99]

2-Butanol	78-92-2		[90]	[91]	[99]
2-Butanone	78-93-3		[90,94-96]	[87,91]	[86,87,90,94,96,99]
2-Butene	624-64-6			[91]	
2-Decanone	693-54-9			[97]	[86]
2-Heptanone	110-43-0	[87]	[96]	[91,96,97]	[86,87,92,99]
2-Hexanone	591-78-6			[97]	[90,99]
2-Methyl-1-propanol	78-83-1			[91]	
2-Methyl-1-propene	115-11-7			[91]	
2-Methyl-2-butene	513-35-9			[91]	
2-Methylnaphthalene	91-57-6		[95]		
2-Methylpyrazine	109-08-0			[97]	
2-Nonanone	821-55-6			[91,97]	[86,87,89,92,98,99]
2-Pentanone	107-87-9			[91]	[86,90,92,99]
2-Pentene	109-68-2				[87]
2-Propanol	67-63-0			[91]	
3-Heptanone	106-35-4				[86]
3-Methyl-1-butanol	123-51-3			[91]	[89,99]
3-Methyl-1-butene	563-45-1			[91]	
3-Methyl-2-butanone	563-80-4				[86,94,99]
3-Methyl-2-pentanone	565-61-7				[86]
3-Methyl-3-buten-2-one	814-78-8				[86]
3-Methyl-3-penten-2-one	565-62-8				[86]
3-Methylbutanal	590-86-3		[85]	[91]	[89,99]
3-Methylbutyl butanoate	106-27-4			[97]	
3-Octanone	106-68-3				[86]
3-Penten-2-one	625-33-2				[89]
4-Ethyl-1,2-dimethylbenzene	934-80-5				[89]
4-Methyl-3-penten-2-one	141-79-7				[89]
5-Methyl-5-hexen-2-one	3240-09-3			[97]	
Acetaldehyde	75-07-0			[91]	[99]

Acetic acid	64-19-7	[87]	[90]		[92,99]
Acetone	67-64-1		[87,90,95,97]		[86,90,93,97,99]
Acetonitrile	75-05-8			[97]	[99]
Acetophenone	98-86-2			[91]	[86,99]
Ammonia	7664-41-7		[90]		[90,93,99]
Benzonitrile	100-47-0			[97]	
Butyric acid	107-92-6		[88,90]		
Carbon disulfide	75-15-0		[90]	[91]	
Decane	124-18-5			[80]	[86]
Diethyl ether	60-29-7				[99]
Dimethyl disulfide	624-92-0		[87]	[91]	[86,90,99]
Dimethyl sulfide	75-18-3			[91]	[86,90,99]
Dimethyl trisulfide	3658-80-8		[87]		[86,99]
Dodecane	112-40-3				[92,93]
Ethanol	64-17-5	[93]	[95]	[91]	[92,99]
Ethyl acetate	141-78-6			[91]	
Ethyl butanoate	105-54-4			[91]	
Ethylbenzene	100-41-4			[97]	
Formaldehyde	50-00-0				[99]
Heptanal	111-71-7				[89]
Hydrogen cyanide	74-90-8				[90,99]
Hydrogen sulfide	7783-06-4		[90]		[90,99]
Indole	120-72-9	[87,88,93,100]			[92,99]
Iso-butane	75-28-5			[91]	
Isoprene	78-79-5	[87]		[87,91]	[99]
Isovaleric acid	503-74-2		[85,88]		
Limonene	138-86-3				[99]
Malondialdehyde	543-78-9				[90]
Mercaptoacetone	24653-75-2			[91]	
Methane	74-82-8		[90]		[90]
Methanethiol	74-93-1			[91]	[99]

Methanol	67-56-1				[99]
Methyl 2-methylbutanoate	868-57-5				[99]
Methyl acetate	79-20-9			[91]	
Methyl isobutyl ketone	108-10-1				[86]
Methyl thiobutanoate	2432-51-1				[99]
Methyl thiocyanate	556-64-9				[89,90,99]
Methyl vinyl ketone	78-94-4			[91]	[86]
Methylbutanal	Not provided				[99]
Methylphenol	Not provided				[99]
Methylthioacetate	1534-08-3				[99]
n-Butanal	123-72-8			[91]	[99]
n-Butyl acetate	123-86-4			[91]	
n-Hexanal	66-25-1				[89,99]
n-Nonane	111-84-2			[91]	
n-Pentane	109-66-0		[90]		[90]
Phenol	108-95-2				[90,99]
Phenylethyl alcohol	60-12-8			[80]	
Propanoic acid	79-09-4				[99]
Pyridine	110-86-1				[89]
Pyrrole	109-97-7				[86]
Terpinen-4-ol	562-74-3				[92]
Toluene	108-88-3				[92,96,99]
Trimethylamine	75-50-3				[88]
Undecane	1120-21-4		[95]		
**Abbreviations:** CAS, Chemical Abstract Service.					
